# Streamlined research funding using short proposals and accelerated peer review: an observational study

**DOI:** 10.1186/s12913-015-0721-7

**Published:** 2015-02-07

**Authors:** Adrian G Barnett, Danielle L Herbert, Megan Campbell, Naomi Daly, Jason A Roberts, Alison Mudge, Nicholas Graves

**Affiliations:** Queensland University of Technology (QUT), 60 Musk Ave, Kelvin Grove, Brisbane, QLD 4059 Australia; Australian Centre for Health Services Innovation (AusHSI), 60 Musk Ave, Kelvin Grove, Brisbane, QLD 4059 Australia; Cerebral Palsy Alliance, 187 Allambie Road, Allambie Heights, Sydney, NSW 2100 Australia; Royal Brisbane and Women’s Hospital (RBWH), Herston, Brisbane, QLD 4029 Australia

## Abstract

**Background:**

Despite the widely recognised importance of sustainable health care systems, health services research remains generally underfunded in Australia. The Australian Centre for Health Services Innovation (AusHSI) is funding health services research in the state of Queensland. AusHSI has developed a streamlined protocol for applying and awarding funding using a short proposal and accelerated peer review.

**Method:**

An observational study of proposals for four health services research funding rounds from May 2012 to November 2013. A short proposal of less than 1,200 words was submitted using a secure web-based portal. The primary outcome measures are: time spent preparing proposals; a simplified scoring of grant proposals (reject, revise or accept for interview) by a scientific review committee; and progressing from submission to funding outcomes within eight weeks. Proposals outside of health services research were deemed ineligible.

**Results:**

There were 228 eligible proposals across 4 funding rounds: from 29% to 79% were shortlisted and 9% to 32% were accepted for interview. Success rates increased from 6% (in 2012) to 16% (in 2013) of eligible proposals. Applicants were notified of the outcomes within two weeks from the interview; which was a maximum of eight weeks after the submission deadline. Applicants spent 7 days on average preparing their proposal. Applicants with a ranking of reject or revise received written feedback and suggested improvements for their proposals, and resubmissions composed one third of the 2013 rounds.

**Conclusions:**

The AusHSI funding scheme is a streamlined application process that has simplified the process of allocating health services research funding for both applicants and peer reviewers. The AusHSI process has minimised the time from submission to notification of funding outcomes.

## Background

The objective of health services research is to improve patient care, improve health-care decision making, and ensure sustainability within healthcare systems. This makes health services research the fundamental research and development arm of the healthcare industry. The importance of health services research is recognised in the UK where funding opportunities are provided by the Department of Health, Policy Research Program [1], and the National Institute of Health Research (NIHR), Health Services and Delivery Research Programme [2]. The funding is substantial and recurring with the NIHR providing £280 million of annual funding for research to improve services. In the USA, funding opportunities are provided through the National Information Center on Health Services Research and Health Care Technology.

Despite the worldwide importance of increased efficiency and sustainability within healthcare systems, health services research is generally underrepresented in Australia compared with funding for the basic sciences [3]. The major funding schemes for health services research are provided by the National Health and Medical Research Council (NHMRC); in 2013, they awarded 5% (AU$42 million) of their annual budget for health services research [3].

The allocation of research funding usually requires peer review to select the most competitive proposals. The scientific community relies on peer review to be robust, fair, transparent and efficient, but these aims may conflict. Funding agencies who manage the process of allocating research funding are requesting increasing volumes of information from applicants in an attempt to optimise fairness and transparency. However, this may impose a significant time burden for applicants, reviewers and administrators.

Australian researchers applying for major NHMRC funding reported spending on average 34 days preparing their applications [4]. In health services research, this time is rarely rewarded with funding from the same scheme, as the success rate for health services research is low; the 2013 funding round was just 13.8% [5]. Compounding this challenge is the desire to engage clinical healthcare professionals in health services research [6], which both ensures alignment of research priorities with important clinical issues and assists with translation of findings directly into practice. However, time constraints for clinical healthcare professionals are pressing.

This paper reports on the development of a streamlined protocol for applying and awarding research funding using a short proposal and accelerated peer review. This streamlined protocol aims to reduce the content and time required by applicants and reviewers and provide rapid and timely decisions on funding outcomes, whilst still providing transparent review with written feedback to assist improved resubmissions. This paper describes the initial experience with this novel protocol and to report against these aims for the first two years of operation.

## Method

### Formation of AusHSI

The Australian Centre for Health Services Innovation (AusHSI) was established in 2011 as a collaborative partnership between Queensland University of Technology (QUT), Queensland Health Office of Health and Medical Research, and the Royal Brisbane and Women’s Hospital (RBWH), the largest public teaching hospital in the state of Queensland. The goal is to support collaborations between healthcare professionals who see the problems in health services and academic researchers who know how to quantify, evaluate and disseminate new ideas, in order to address pressing health system challenges. AusHSI consists of an academic team (academic director, statistician, centre manager and administrative support) from QUT, and three part-time clinical directors representing medicine, nursing and allied health professions from RBWH, who act under the direction of a management committee representing the collaborative funding partners.

### AusHSI funding scheme

AusHSI Stimulus Grants are for applied research about health services challenges, and are currently only available to healthcare professionals in the state of Queensland. Successful applicants are awarded up to AU$80,000 for a maximum 12 month project. To determine eligible proposals, the AusHSI definition of health services research is the examination of the funding, organisation and delivery of health services from multidisciplinary perspectives. The research outcomes are usually at the population level rather than the individual; this focus contrasts with clinical research which emphasises individuals.

The two criteria for AusHSI funding are that the research team represents a good partnership between a healthcare professional and a full-time researcher, and the outcomes will lead to rapid and large improvements in health services. The applicants’ track record is not a major consideration. Prior to the opening of the funding round, AusHSI provides web-based and face-to-face seminars to inform potential applicants about the requirements for a strong proposal. The funding policy is available from the AusHSI website: www.aushsi.org.au.

### Streamlined proposals

A streamlined proposal is used to minimise the time spent preparing and reviewing grant proposals; evidence of track record is not required. Each funding round is open for four weeks. Applicants are asked to write about their partnership, research question, method, budget and expected improvements to health services within the 1,200-word limit. They submit their proposal using a secure web-based portal.

### Accelerated peer review

The accelerated peer review process is conducted by the AusHSI Scientific Review Committee (SRC, Table 1). Initially, two SRC members independently categorise the proposals as “reject”, “revise” or “accept for interview” and provide written feedback using a secure web-based portal. The SRC is convened to discuss and reach consensus for proposals where one or both independent reviewers categorised the proposal as “accept for interview”. This discussion is summarised as additional feedback. The SRC finalises the proposals to be invited for interview. The interviews occur within 10 days of shortlisting. Applicants present to the SRC for 10 minutes with an extra 10 minutes for questions, the applicant departs, and there is 10 minutes of SRC discussion. The proposal is given a rank rather than a score. Rank is determined according to the key criteria of feasibility (including time lines and the skill mix of the research team); the study design, with a preference for simple study designs using high quality data; and the impact on health services (including the potential cost savings and improvement to patients’ lives). Funding is allocated from the highest ranked proposal down until the pre-defined budget limit for the round is met. Successful applicants are notified within two weeks of the interview.

**Table 1:** **Membership of the AusHSI scientific review committee**

**Member**	**Role description**
AusHSI Academic Director	Chair of the committee and ensures the shortlisted proposals align with the strategic directions for health services research, and as an expert advisor.
(1 person)	
Clinical Directors	Expert advisors in the disciplines of Medicine, Nursing and Allied Health.
(3 persons)	
Statistician	Reviews every application as an expert advisor on the proposed methods and data analysis.
(1 person)	
Ethics specialist	External health research ethics specialist to identify any ethical concerns.
(1 person)	
External expert advisors	Advisors whose membership rotates between funding rounds, including an expert in qualitative analysis.
(3 persons)	

AusHSI: Australian centre for health services innovation.

Applicants with a final ranking of ‘reject’ or ‘revise’ receive written feedback and suggested improvements for their proposals within three weeks of the finalised outcomes. All applicants receive feedback that summarises the initial reviews and any discussions during shortlisting meetings. To increase transparency of the peer review process, the SRC discussion after the interview was audio-recorded and transcribed for Round 1–2013. Interviewed applicants were sent an edited transcript of the discussion of their proposal. At Round 2–2013, the SRC discussions were audio-recorded and reviewed to enhance the feedback provided in the written summaries; transcripts were not provided for this funding round due to time constraints.

### Conflict of interest

In recognition of the small community of researchers in the state of Queensland, AusHSI deals with conflict of interest (COI) in a consistent, transparent and rigorous manner. The Australian Code for the Responsible Conduct of Research stipulates that participants in peer review should: be fair and timely in their review; act in confidence and not disclose the content or outcome of any process in which they are involved; and declare all COI [7]. AusHSI adheres to this code and a COI is declared in situations in which the SRC member has an interest, which may have influenced, or be perceived to influence, the proper performance of the member’s responsibilities in reviewing the proposals. The perception of a COI is as important as any actual COI, and may be declared at any stage of the peer review process if new conflicts become apparent.

### Getting good value from proposals

AusHSI is eager to build capacity in research among healthcare professionals and unsuccessful applicants are strongly encouraged to improve their proposal according to the feedback provided to them, and then resubmit in future funding rounds. Applicants are asked to identify their proposal as a resubmission from the previous round by providing the original application number. The SRC specifically checks resubmissions for evidence of revisions based on the feedback for the original proposal. Proposals that are shortlisted but not funded due to budget allocations can be categorised as “near-miss”. If requested by the applicant, AusHSI will provide a letter of support that may help gain funding from other schemes.

### Descriptive evaluation

Four AusHSI funding rounds are summarised in Table 2. For the purpose of this evaluation, applicants were asked to estimate the number of days they spent preparing their proposal, and the time from submission to notification of funding decision is recorded for each round. Applicants were invited to respond to their written feedback using email; these responses have been summarised without a formal qualitative analysis.

**Table 2:** **Summary statistics from AusHSI stimulus grant funding rounds (2012–2013)**

**Timeline**	**2012**		**2013**	
	**Round 1**	**Round 2**	**Round 1**	**Round 2**
	**May–Jun**	**Aug–Nov**	**Apr–Jun**	**Aug–Nov**
	**n (%)**	**n (%)**	**n (%)**	**n (%)**
Applications received	111	108	46	39
Eligible applications	74	89	34	31
Resubmissions	–	15 (17)	12 (35)	11 (35)
Shortlisted	29 (39)	26 (29)	27 (79)	22 (71)
Interviewed	11 (15)	8 (9)	11 (32)	10 (32)
Funded after interview	6 (8)	5 (6)	5 (15)	5 (16)
**Applicants’ time spent on proposal**				
Mean days	6.8	7.2	7.2	7.7
Median days (min – max)	5 (1 – 31)	5 (1 –30)	5 (1 – 30)	4 (1 – 48)
**Administration**				
Submission to notification	6 weeks	8 weeks	7 weeks	8 weeks
Allocated funding (AU$)	275,000	299,756	300,000	330,000
Median budget (AU$)	70,626	75,494	66,237	60,000

AusHSI: Australian centre for health services innovation.

This original data was collected as part of a quality improvement evaluation that did not require ethics approval or the consent of applicants. This observational study used this existing data that was non-identifiable data about human beings and was confirmed as exempt from the need for University Human Research Ethics Committee at the Queensland University of Technology (exemption number 1400000998). This is in-line with section 5.1.22 of the guidelines from the Australian National Health and Medical Research Council [8].

## Results

### Funding outcomes

AusHSI has held four funding rounds from May 2012 to November 2013 (Table 2), and provided funding for 21 research projects. The total number of received and eligible proposals decreased over the four funding rounds. As the number of eligible proposals decreased from 2012 to 2013 (n = 74; n = 89; n = 34; n = 31), the SRC shortlisted an increasing proportion for discussion; but the absolute number remained similar (from 29 to 22 proposals). The proportion of proposals to succeed in obtaining funding increased from 6% (in 2012) to 16% (in 2013) as the number of eligible proposals decreased.

Applicants spent on average seven days preparing their proposals. Peer reviewers spent on average 36 minutes (range 15–105 min) assessing each proposal prior to the face-to-face panel meeting where the same reviewers spent 10 min discussing each proposal. Successful research teams were notified within two weeks of interview, which was a maximum of eight weeks after the submission of their proposals. The proportion of resubmissions represented 35% of eligible applications in the 2013 rounds of funding. The broad scope of health services research is demonstrated by the funded proposals (Table 3).

**Table 3:** **Funded AusHSI stimulus grant proposals, by health services**

	**2012**		**2013**	
**Health services in proposal**	**Round 1**	**Round 2**	**Round 1**	**Round 2**
Chronic disease				1
Clinical practice	1	1		1
Emergency department			1	1
Mental health	2		1	
Musculoskeletal health		1		
Nutrition			1	
Oncology				1
Oral health	1			
Pathology & pharmacology			1	1
Patient safety		1		
Surgical practice	1	1	1	
Telehealth	1	1		
**Total funded**	**6**	**5**	**5**	**5**

### Short proposals

Many applicants reported their appreciation of the “simple online application process” and having instructions that were “clear and straightforward”. The development of clear guidelines and definition of health services research ensured the proposals were tailored to AusHSI’s strategic directions and reduced the number of ineligible proposals submitted in 2013 (Table 2). Applicants “found the website very helpful” and “the web based seminar was a great help” in preparation of their proposals. The 1,200-word limit for a proposal was found to be “challenging but not impossible” and “reduces a lot of the unnecessary paperwork” encountered in other funding schemes. One applicant reported the “focus on a good research idea rather than research track record, is refreshingly different and novel”.

### Feedback to applicants

Providing comprehensive written feedback to applicants is a key strategy of the AusHSI funding scheme to further develop the skills of health services researchers. AusHSI provides feedback to applicants based on the: 1) comments from two SRC members; 2) summary of the SRC discussion at shortlisting; and 3) summary of post-interview SRC discussion. Some applicants, including those who were not funded, provided feedback on the streamlined protocol.

Applicants reported that they appreciated receiving “quick feedback” on their proposal because it was “helpful in refining the proposal [and] provided encouragement to the team to resubmit”. Applicants, regardless of their funding success or failure, found the process provided them the opportunity to “learn and create better research applications” and complete “proof of concepts” from which to build their potential for applying for larger funding schemes. Interviewed applicants reported the transcripts were “incredibly helpful” and would be used “to improve my project on a larger scale”.

## Discussion

### Application numbers

The decrease in the number of applications over time (Table 2) was in part due to a reduction in the number of ineligible applications. This is because AusHSI was a new initiative and it took time to establish an understanding with the research community about AusHSI’s goals and what research we aimed to support. Another potential reason for the decrease was the relatively small pool of health services researchers in Queensland. This meant we initially received a large number of unfunded ideas, some of which we were able to support. One of AusHSI’s key goals is to increase research capacity in health services research so that good ideas continue to be generated.

### Streamlined research funding

A streamlined protocol for applying and awarding research funding has been successfully developed using a short proposal and accelerated peer review scheme. AusHSI has used this protocol for four funding rounds within 18 months and awarded a total of 21 grants with a total budget of AU$1.2 million. Applicant and reviewer time commitments were relatively modest, and successful applicants were notified within eight weeks of submitting their proposal.

There is substantial current interest in streamlining research processes. In 2013, the Canadian Institute of Health Research (CIHR) changed their peer review processes in recognition of the need to minimise the applicant and reviewer burden from each step of their funding schemes [9]. In Australia, the 2013 review into the NHMRC funding schemes has recommended streamlining grant proposals [6]. Reform to grant funding processes by using streamlined protocols reduces the costs to applicants, reviewers and administrators.

The AusHSI timeline is comparable to a two month turnaround at the CIHR for health services research [10], but shorter than the UK funding schemes for health services research where the time from submission to notification of outcomes ranges from 4–5 months [1], or longer when using an initial proposal and subsequent invitation to submit a full application within eight weeks followed by further peer review [2]. The AusHSI funding scheme demonstrates that a streamlined process is feasible, and similar processes have already been adopted by other funding schemes within the Queensland Government [11].

Choosing who to fund often tends to rely on the track record of the researchers [12]. Evidence of prior research success might predict future success, but it might not. It is systematically irregular to reward what people have done in the past when the relevant question is what they are about to do. This is particularly important for health services research which is a developing field with many new investigators. However, track record may be used to provide a proxy measure for feasibility of the current proposal and offset the risk aversion of the peer reviewers.

### Comprehensive feedback

Innovative reforms such as providing comprehensive feedback to applicants makes use of the wealth of information collected during peer review. An irony of peer review for grant funding is that large costs are incurred collecting information that will enable applicants to improve their research, for example many experts on review panels pick apart the minutiae of proposals and discuss the problems, yet for some schemes the information is not provided to the applicants at all, or a minimum version is provided.

The UK Engineering and Physical Sciences Research Council survey on peer review acknowledged the need to maximise the use of the collected information, and provided recommendations on the importance of feedback to applicants [13]. Unfortunately, providing transparent feedback is rare and most funding peer review processes remain hidden [14]. These administrative practices may reflect an underlying risk aversion to potentially receiving formal complaints from the applicants.

Following an unsuccessful funding outcome, many researchers will refine and resubmit their proposals in future funding rounds. By explicitly identifying these proposals as a re-submission and providing information generated from the original peer review into the current re-review, AusHSI maximises the use of all available information and provides research groups the opportunity to respond constructively to feedback. The high proportion (35%) of resubmissions in later rounds attests to the value placed by researchers on this process. In comparison, the NHMRC provides limited feedback to researchers with a score and single comment, and this lack of feedback offers no assistance in improving applications for later submissions.

### Applicability at larger scale

A potential limitation of the AusHSI streamlined protocol is that the process may not work on a large scale. In 2013, the NHMRC administered the peer review of 3,821 Project Grant applications but only 145 of the applications were for health services research [5]. AusHSI received around 75% of this volume of applications for each 2012 funding round, despite being limited to the state of Queensland (Table 2). However, with AusHSI’s smaller funding budget, the proposals have more limited scope than typical NHMRC applications. A practical upper limit for streamlined funding schemes is yet to be identified for the AusHSI process because the 2013 funding rounds received fewer proposals than in 2012.

The AusHSI system of detailed feedback could be further refined to only those applicants with ‘near–miss’ proposals, i.e., highly ranked but not funded. These researchers would be encouraged to explicitly address the detailed comments when re-submitting these proposals. The US National Institutes of Health (NIH) currently use such a system for the re-submission of applications; allowing only one resubmission within 37 months of the initial proposal, the aim of which is “to facilitate funding of high quality applications earlier, with fewer resubmissions” [15]. The NIH aims to make the best use of all information from the prior peer review as part of the current re-review.

### Alternative funding models

The funding opportunities for health services research in Australia are spread among government and non-government organisations. Beyond the larger NHMRC funding, there is no central agency to administer the smaller grants available to health services researchers (such as those made available through state governments, hospitals, and professional organisations). In the absence of a central agency, individual organisations establish their own processes. For example, the Cancer Council have adapted the Delphi process, used in clinical settings, to award funding in an efficient, transparent, equitable and reproducible system [16].

A recommendation to build capacity in health services research by establishing a national institute was stated in the 2013 review of the NHMRC funding schemes [6]. It is possible a health services research institute could play an influential role in the allocation of future centralised funding for innovative proposals in Australia, including an efficient, transparent and responsive system for grant review and funding allocations.

## Conclusion

The AusHSI funding scheme uses a streamlined application process that minimises the burden of grant applications on both applicants and reviewers, and provides a short eight week turnaround from submission to notification of funding outcomes. Prompt comprehensive feedback provides researchers the opportunity to resubmit improved proposals. The feedback to applicants contributed to fewer, better quality proposals as the streamlined protocol is developed, further improving efficiency for both applicants and reviewers.
